# Risky outdoor play in the early years: how are parental and practitioner perceptions of danger and benefits associated with young children's outdoor play experiences?

**DOI:** 10.1080/21594937.2025.2508649

**Published:** 2025-07-04

**Authors:** Paul McCrorie, Avril Johnstone, Natalie Nicholls, Marine Keime, Boris Jidovtseff, Anne Martin

**Affiliations:** a MRC/CSO Social and Public Health Sciences Unit, School of Health and Wellbeing, University of Glasgow, Glasgow, UK; b Department of Sport and Rehabilitation Sciences, Research Unit for a life-Course perspective on Health and Education, University of Liege, Liege, Belgium

**Keywords:** Risky play, parent, practitioner, perceptions, early years, childcare

## Abstract

Against a backdrop of generational declines in outdoor play and recent work demonstrating geo-cultural differences in adult perceptions of risk in outdoor play, this paper explores the relationships between gatekeeper (parent/practitioner) perceptions of dangers and benefits in outdoor risky play scenarios and young children’s (aged 2–5 years old) experiences of outdoor play in Scotland, and whether these are moderated by being a parent or practitioner. A contextually relevant picture-based survey asked parents (*n* = 205) and practitioners (*n* = 151) to rate six different outdoor risky play scenarios based on their level of danger and benefits. The outcome variable was number of outdoor play scenarios children experienced. Ordinal logistic regression analyses were supported by the variables: respondent age, respondent experiences as a child, child age, perceptions of road traffic, and urbanicity of home/practitioner setting. Results suggested that an increasing number of perceived dangers was generally reflected in lower odds of the children having play experiences. This effect was consistent regardless of being a parent or practitioner. When perceived benefits were included in the model the effects of perceived danger became non-significant. Contributing Scottish evidence to the wider literature supports context specific intervention efforts promoting the benefits of outdoor (risky) play.

## 1. Introduction

‘Seven percent of childhood accidents start with jumping’ according to actor Hugh Bonneville’s anxious and risk averse parent character Henry Brown, talking at his young son as he jumps up and off a flat bench in the opening scene of the 2014 screenplay of everyone’s favourite bear, and UNICEFs Champion for Children, ‘Paddington’. A child’s early experiences of outdoor play are largely dependent on the perceptions, attitudes, and behaviours of adult gatekeepers, such as parents, caregivers or early learning and childcare (ELC) practitioners (Cheng et al., 2022; Jerebine et al., 2022a, 2022b; Loebach et al., 2021; Sandseter et al., 2020). As we see in Paddington, these adult gatekeepers can either afford or constrain a child’s opportunity to engage in outdoor risky play (Jerebine et al., 2022a, 2022b).

Although a provocative topic as it relates to child safety, (Brussoni et al., 2012; Little et al., 2012), engaging in outdoor risky play has been shown to provide opportunities for children to explore and understand the world. Moreover, children have a unique ability to progressively increase the level of difficulty of a task (Kleppe et al., 2017), exerting control and management over the experienced risk of a situation, challenge and test their physical and psychological limits, explore boundaries, increase their confidence, and learn/develop new skills (Brussoni et al., 2017; Fjørtoft, 2004; Lavrysen et al., 2017; Little & Wyver, 2008). Even with these reported positive associations as a backdrop, we continue to see inter-generational decline in outdoor risky play (Kyttä et al., 2015; Witten et al., 2013), with present generations of children more likely to spend time indoors sedentary playing virtually (Dodd et al., 2021; Kemple et al., 2016). Geo-cultural differences in risk perception are evident; some early childhood practices across the Scandinavian countries for instance are viewed, and legislatively supported, as sensible and normalised childcare, yet considered risky by many in other countries (New et al., 2005). As such, it can be argued that the understanding of the factors involved in increasing children’s exposure to risky play opportunities is both culturally and contextually specific, yet limited research exists in the UK exploring these important factors in parents, caregivers and Early Learning and Childcare (ELC) practitioners, with some of the most recent evidence coming from older children (Dodd et al., 2021). Northern/central European countries may not truly reflect the geographical and cultural context of the UK. Moreover, with significant institutional, political, and economic differences within the UK because of devolved powers to the Home Nation’s Executive (i.e. the Scottish Government), certain policy and legislative areas, such as Health, Education and Training are implemented differentially across the UK, strengthening the argument to explore issues around adult barriers to risky outdoor play in Scotland.

To fully consider the extent of children’s experiences with risky play opportunities, it is valuable to consider the daily settings children are exposed. Two of the most important being the home and ELC setting environment. In both settings, it is usually the parent/guardian and ELC practitioners, respectively, who acts as the primary gatekeeper to experiencing outdoor risky play opportunities for the child (Adams, 1995). As such, appreciating and understanding the underlying, contextually and culturally fixed, factors associated with parental and practitioner attitudes is vitally important if we are to reverse the negative trends in children’s outdoor play.

## 2. Context for the research

Engaging in outdoor play in early childhood is crucial for children’s physical, social and emotional development (Brussoni et al., 2015; Johnstone et al., 2022). The strongest evidence exists primarily in relation to physical activity, where several systematic reviews highlight that a greater time spent in outdoor play is associated with higher levels of physical activity in childhood (Brussoni et al., 2015; Gray et al., 2015; Lee et al., 2021; Martin et al., 2022; Truelove et al., 2018); with some longitudinal evidence suggesting this may also persist into adulthood (Telama et al., 2014). Outdoor play is also important for social and emotional development: it promotes opportunities for interactions with peers and other adult gatekeepers; its unstructured nature enables children to practice communication, empathy and sharing; and it provides a sense of freedom that encourages resilience and reduces stress and anxiety (Burdette & Whitaker, 2005; Rademacher & Koglin, 2018). Given that the early years is a critical time where habits and behaviours are established, accompanied by rapid improvement in children’s development (Pearce et al., 2020), it is important that opportunities for outdoor play are provided from an early age.

One subcategory of outdoor play, namely risky play, continues to receive notable levels of academic and media/public attention (Lee et al., 2022; Sandseter, 2009). Children’s risk taking in play contains a set of common characteristics, including curiosity, exploration, deep concentration, fear and excitement (Kleppe et al., 2017), and Sandseter et al. (2020, p. 22) further defines risky play as ‘thrilling and exciting forms of physical play that involve uncertainty and a risk of physical injury’ (Sandseter, 2010). Indeed, children actively search for opportunities to engage in risky play, driven by the feelings of exhilaration that border on fear (Sandseter, 2009). This working definition has been operationalised to reflect six main categories (Sandseter, 2010): (i) play with *great heights* (danger of injury or falling); (ii) play with *high (uncontrolled) speed* (danger of collision); (iii) play with *dangerous tools* (e.g. saw, axe that can lead to injury); (iv) play near *dangerous elements* (e.g. fire pit, water); (v) *rough-and-tumble* play (danger of children harming each other); (vi) play where the children can *disappear/get lost* (e.g. unsupervised near woods).

However, the genuine concern for the physical and emotional safety of children over the last 30 years has been accompanied with the over-protection from, and over-legislation/regulation of, risk from the lives of children in western countries such as the US, Canada and the UK (Ball, 2004; Batty, 2010). Fuelled in part by the fear of litigation (Ball, 2004) and propagation – and distortion – of risk (e.g. stranger danger or neighbourhood crime/ traffic safety) by the mass media, generational shifts in gatekeeper’s decision making has been shown to influence things like children’s experiences of playing in inclement weather conditions (New et al., 2005), in playgrounds (Ball, 2004) on streets (Witten et al., 2013), and their independent mobility (McGinn et al., 2008). Evidence from Jidovtseff and colleagues in Belgium has demonstrated that parental assessment of the benefits and dangers with certain types of risky play is associated with whether children are given permission to engage in those activities (Jidovtseff et al., 2022). Perceived benefits appeared to have a stronger influence than the perceived dangers, and parent’s own childhood experiences were significantly associated with their perceptions of benefits, dangers, and their children’s reported experience of different types of risky play scenarios. For this paper we borrow the methodological approach as reported by Jidovtseff et al. (2022) and apply similar processes in a Scottish population – with two main adaptations: (i) young children’s opportunities to experience risky play is not only dependent on parents but also on practitioners and/or childminders who provide care within ELC settings. As such we wanted to expand our analyses to include practitioner perceptions and compare these to parents to see if they were more or less liberal; and (ii) whereas the central focus of the analysis by Jidovtseff and colleagues was the predictive properties of parents’ perceived danger, benefits, and competence (of the chid) on the level of parental permission to play outdoors, the analysis underpinning this paper uses the children’s actual experience of risky play (as reported by their parent/guardian or practitioner) as the main outcome of interest. These questions are broken down further to:

1. Is perception of danger negatively associated with perceived benefits? Is this relationship influenced by whether the respondent is a parent or practitioner?
2. Is the number of perceived benefits or dangers associated with being a parent or a practitioner?
3. Is there an association between perceptions of danger and benefits in outdoor play and children’s experience of risky outdoor play? Is this relationship moderated by:
  - Child age
  - Age of respondent
  - Whether the respondent is a parent or practitioner?

## 3. Materials and methods

### 3.1. Photo-based questionnaire

Photo-based research has been prevalent across many academic fields (e.g. Jacobsen, 2007). Studies involving the perceptions of place, landscapes, and outdoor scenery, for instance, have had to rely on still photographs, frequently justified as an alternative to excessive costs associated with bringing participants to sites to experience in person (Daniel, 2001). A recent such example exploring outdoor risky play was conducted by colleagues in Belgium (Jidovtseff et al., 2022) from which this study borrowed the methodology and procedure and adapted it to the Scottish context.

In short, two photo-based surveys – one for parents and one for educators in early learning and childcare – were developed in French, translated to English, and administered to parents of children aged 2–5 years old and practitioners of ELC centres responsible for children aged 2–5 years old.

#### 3.1.1. Questionnaire development – parent survey

The initial parent questionnaire, developed by Jidovtseff and colleagues, contained ten items/situations: six of these represented the main risky play categories identified by the initial work of Sandseter (2007), whilst four further situations were included by the Belgian team to reflect other outdoor play situations requiring parental permission, which carry some degree of perceived/real danger: (i) meeting animals (being bitten), (ii) discovering (in)edible plants and berries (being poisoned); (iii) playing barefoot (standing on broken glass, needles, being burned); and (iv) playing in inclement weather conditions (getting sick).

The pictures had to represent the reality of the situation (i.e. the risk associated) and visually demonstrate both risk and benefit. The age of the children within the pictures had to mirror the target age group and had to include both boys and girls. Additionally, it was important to avoid floor or ceiling effects. As such, the chosen pictures could not be perceived as too risky or not risky at all.

#### 3.1.2. Questionnaire structure

For each situation, participants were questioned on six main dimensions: perception of dangers (Q1); perception of benefits (Q2); perception of child competence (Q3); parental permission to play (Q4), child's previous experience in the presented situation (Q5); and parents’ previous experience when a child (Q6). Each situation was accompanied by the same questions (Table 1). Three questions had a filter function (Q1, Q2 and Q4). For example, if parents scored a situation as dangerous in Q1 (either rather agree or completely agree), they were asked the following additional question: ‘What danger(s) are you most concerned about in this situation?’ (Q1b). They were asked to select potential perceived dangers from a predefined list of answers. At the end of this list, they were given the opportunity to add any other perceived danger that was not included in the predefined list. A similar follow up question was used for the perception of benefits (Q2 – naming the benefits) and permission to play (Q4 – naming the conditions where they would allow permission).

**Table 1.** Survey questions, measured dimensions, and scale responses.

		Questions			Answers		
Measured dimensions	Parents	Practitioners					
(Q1) Perceived benefits	This activity seems beneficial for my child (Q1)*	This activity seems beneficial (Q1)*	Strongly disagree	Rather disagree	Neither agree nor Disagree	Rather agree	Completely agree
(Q2) Perceived dangers	This is a dangerous situation for my child (Q2)*	This is a dangerous situation (Q2)*	Strongly disagree	Rather disagree	Neither agree nor Disagree	Rather agree	Completely agree
(Q3) Perceived competence	My child is physically able to carry out this activity (Q3)	N/A	Strongly disagree	Rather disagree	Neither agree nor Disagree	Rather agree	Completely agree
(Q4) Permission to play	Would you give permission to your child to do this activity? (Q4)*	Would you allow the children do this activity? (Q4)*	Never, under no conditions	Yes, in few cases and under certain conditions	Yes, in most cases and under certain conditions	Yes always	
(Q5) Child experience	Has your child ever experienced this kind of situation? (Q5)	Have the children ever experienced this kind of situation in your early learning centre? (Q5)	No, they have never experienced this situation	I am not sure, I don’t remember.	Yes, they have experience this situation occasionally	Yes, they have experienced this situation many times	
(Q6)Parent experience	Did you experience this type of activity when you were approximately your child's age? (Q6)	Did you have the opportunity to carry out this type of activity when you were approximately the age of the children? (Q6)	No, I have never experienced this situation	I am not sure, I don’t remember.	Yes, I have experienced this situation occasionally	Yes, I have experienced this situation many times	

* Answers with follow-up questions.

#### 3.1.3. Direct translation, validation and ‘Think aloud’ technique

We adapted the original survey to the Scottish context by using the ‘Think Aloud’ protocol (TAP) (Charters, 2003). The parents (100% women) took part in an online Zoom (Zoom Video Communications, inc) interview, where they completed the questionnaire, voicing any concerns and ambiguities as they proceeded. Subsequent survey adaptations included matching the visual presentation of the risk in question with accompanying text (i.e. ensuring the text that accompanied the picture was descriptive but specific to the situation represented by the associated picture); adding ‘neutral’ options across Q1-Q3 (‘neither agree nor disagree’); changes in language where ambiguity existed; and adding in an option ‘I am not sure, I don’t remember’ in reference to Q5 and Q6. See Table 1 for detail of final scale response options.

### 3.2. Survey development – practitioners

Like the parent survey, the practitioner survey adapted one that was originally developed by the team in Belgium. The structure and questions were similar in format with a few differences to reflect the context of the ELC setting: (i) The practitioner survey used in this study had two different versions, split by age group – one for practitioners responsible for children aged 24–36 months (2–3 years) and the second for practitioners responsible for children aged between 37 and 60 months (3–5 years); and (ii) Both versions contained ten different pictorial representations of outdoor activities that children within an early learning and childcare setting may encounter. Using the findings from the think aloud process, changes were made to edit the text in both parent and practitioner surveys to reflect the terminology used in a Scottish context (e.g. changing ‘supervise’ to ‘responsible for’ and ‘hosting environment’ to ‘early learning centre’).

### 3.3. Participants – main survey

Parents were recruited using national parenting organisations social media pages (e.g. Facebook). The parental survey was hosted on the LimeSurvey platform (https://www.limesurvey.org/en/). Parents completed the survey between October and December 2020. To be eligible for the survey, participants had to be parents of at least one child aged 2–5 years and reside in Scotland. If parents had multiple children within this age range, they were asked to choose a child at random to answer questions against.

Practitioners were recruited through Early Years Scotland, a national support organisation with professional membership. The practitioner survey was hosted on the REDCap (https://www.project-redcap.org/). Practitioners completed the survey between September and November 2020. Practitioners were asked to answer questions broadly across the age group they were responsible for rather than for any individual child. Ethical approval was granted by the University of Glasgow’s College of Social Sciences Research Ethics Committee (Ref: 400200017).

### 3.4. Approach to analysis

#### 3.4.1. Harmonisation of parent and practitioner datasets

In total, there were six comparable scenarios that explored the same underlying play construct: (i) Play at great heights, (ii) with high speed; (iii) near dangerous elements (water); (iv) rough and tumble play (fighting with sticks) (v) play with animals; and (vi) play in inclement weather.

#### 3.4.2. Outcome variables

The primary outcome variable of interest was the number of outdoor risky play activities experienced by the children. From Table 1, where answers to the question for each scenario were in the affirmative, i.e. ‘yes, many times’ and ‘yes, occasionally’, these were scored as a one; both ‘I am not sure’ and ‘No, never experienced … ’ were scored as a zero. The number of outdoor risky play activities experienced were then summed out of a total of six. Descriptive analyses indicated that over 70% of children were reported to have experienced five or six. As such, the outcome was regrouped into an ordinal scale: four experiences or fewer (29% of responses), five experiences (28%) and six experiences (43%).

#### 3.4.3. Independent variables

The main variables of interest were parent/practitioner perceptions of danger and benefits in the outdoor play scenarios, represented by the number of scenarios parents/practitioners either rather agreed or completely agreed with the statement that the scenario depicted in the pictures was dangerous or beneficial – the highest total could be six (i.e. respondent rather agreed or completely agreed with each of the six statements). Descriptive analyses suggested approximately 57% of participants perceived zero or one scenario to be dangerous, with almost 76% of participants finding benefit in five or six of the play activities. ‘Perceived Danger’ was regrouped into four categories: none (30%); one (28%); two (21%); three or more (21%). Similarly, ‘Perceived benefits’ was regrouped into: four or fewer (24%); five (29%); and six (48%) to reflect a cell size greater than 20%. These allowed us to retain substantive qualitative variation across the scale that had meaningful categories, whilst maximising statistical power, and reducing the potential for inflated standard errors.

#### 3.4.4. Covariates and confounding

Multiple variables were included to control for confounding, including parent or practitioner (binary 0/1), respondent age (continuous variable in years), child age (two level variable categorising age as: 24–36 months and 37–60 months), respondents’ own personal experience of the play scenarios (total number of scenarios experienced as a child: ‘three or fewer’, ‘four’, ‘five’, or ‘six’), perceptions of road traffic around the home/ELC setting (four-point Likert scale ranging from ‘not dangerous at all’ to ‘very dangerous’), and a binary variable that recognised the home or ELC setting to be either within an urban or rural location (using the two-fold Scottish classification; Scottish Government, 2022).

#### 3.4.5. Statistical analysis

All statistical analyses were conducted in Stata (v17.0). Descriptives, including participant characteristics were analysed using means and standard deviations or medians and IQR. Appropriate analyses were conducted to address each of the stated research questions of the paper. RQ1 employed Kendall’s tau-b (*T*_b_) correlation coefficient, a nonparametric measure of the strength and direction of association between the two ordinal variables perceived danger and perceived benefits. We stratified by respondent (parent or guardian) to answer the sub-question of RQ1. A 3 × 4 (perceived benefits x perceived dangers) contingency table also compared the observed number of respondents within each cell against the expected number and was tested using chi-square. RQ2 used chi-square to test the association between the number of situations experienced by the children and parent/practitioner perceived benefits and dangers. With the outcome variable being ordinal, RQ3 employed a series of ordinal logistic regression using maximum likelihood estimation in the *ologit* package in Stata. The proportionality of odds/parallel regression assumption was checked using the *omodel* package (Wolfe, 1997) and generalised ordered logistic regression, employing the *gologit2* package was used when this assumption was violated (Williams, 2006). Factor variables were tested (that all coefficients were equal to zero) for significance using Wald tests and odds ratios presented for interpretation. To answer RQ3, we tested a core set of regression models:

1. Base Model exploring: The main effects of perceived danger on children’s experience of risky play, with the core set of variables: parent/practitioner, age of respondent, age of child(ren), respondent’s own experience of risky play.
2. Addition of ‘perceived benefits’
3. Addition of ‘environmental’ variables: urban/rural; perceived traffic within area.
4. Separate models exploring Interaction effects of perceived dangers with perceived benefits; child age; respondent age; and whether respondent was a parent or practitioner.
5. Two further models exploring the interactions between perceived benefits and (i) child age; and (ii) age of the respondent.

## 4. Results

The sample was approximately 93% female with practitioners of significantly older median age than parents (median age of the practitioners v parents: 48 years old v 37 years old, *p* < 0.001). There was an even distribution of child ages represented across both parents and practitioners, although a greater proportion of parents lived in urban areas compared to practitioners (χ^2^ = 9.3, *p* = 0.002) (See Table 2).

**Table 2.** Sample demographics and descriptive statistics combined and stratified by respondent groupings.

		Parent (n = 205)	Practitioner (n = 151)	Total (n = 356)
	**^±^**Age (years)	37 (33-40)*	48 (39-54)	39 (34-47)
Gender	Female	179 (87%)	149 (99%)	328 (92%)
Age of child	2–3 years	58 (29%)	45 (30%)	103 (30%)
	3–5 years	140 (71%)	106 (70%)	246 (70%)
Location	Urban	119 (58%)*	63 (42%)	182 (51%)
	Rural	86 (42%)	88 (58%)	174 (49%)
				
	^≠^Scenarios experienced (respondent)	5 (4-6)	6 (5-6)	5 (4-6)
	^≠*^Scenarios experienced (child)	5 (5-6)	5 (3-6)	5 (4-6)
				
	^≠^Number of dangers	1 (0-2)	1 (0-2)	1 (0-2)
	^≠^Number of Benefits	6 (5-6)	5 (5-6)	5 (5-6)
				

**^±^** Median and IQR; practitioner *n* = 147.

≠ Median (IQR).

*P < 0.01, parent vs practitioner representation.

¥ Percentage of parent and practitioner sample who perceived both benefit and danger in each play situation.

No significant differences were found between parents or practitioners for reported number of benefits (χ^2^ = 1.97, *p* = 0.37) or dangers (χ^2^ = 1.83, *p* = 0.60). Practitioners were, however, more likely to report a higher number of personal play experiences as a child (χ^2^ = 21.5, *p* < 0.001), but lower number of play experiences for the children under their care (χ^2^ = 22.3, *p* < 0.001).

Both groups of participants recognised that each scenario held benefits and/or dangers. For example, 33% of participants perceived danger and no benefit with playing with dogs, followed by play fighting with sticks (22%), playing at high heights (13%), water (10%), speed (8%), and rain (3%). Over 90% of participants recognised the benefit and no danger of playing in the rain, followed by speed (84%), playfighting (71%), height (65%), water (54%), and animals (48%). Finally, 36% of participants perceived both benefit and danger associated with playing near water, followed by playing at high height (23%), with animals (19%), at speed (9%), playfighting (8%), and in the rain (4%). Parents were more likely to identify dangers in play scenarios than practitioners,

In general, higher numbers of perceived benefits were associated with fewer perceived dangers (τ_b_ = −0.27, *p* < 0.001); similarly demonstrated when these variables were reduced to three categories (χ^2^ = 39.6, *p* < 0.001). This relationship held true for both parents (τ_b_ = −0.27, *p* < 0.001) and practitioners (τ_b_ = −0.26, *p* < 0.001). In other bivariate tests, the number of play situations experienced by children was associated with both the number of participant perceived benefits, and the number of perceived dangers. Fewer perceived total benefits (χ^2^ = 47.9, *p* < 0.001) and more dangers (χ^2^ = 32.4, *p* < 0.001) were associated with fewer experiences for/by the children.

For overarching Research Questions 3a-c, no moderating effects (steps 4 and 5 of the regression modelling; by respondent group; respondent age; child age group; perceived benefits) were found and so the following represents main effects only. Across all analyses, the assumption of proportionality of odds across the response categories was confirmed and as such ordinal logistic regression used. In step 1 of the modelling (base model), perceived danger was a significant main effect (Wald test; χ^2^ = 14.5, *p* = 0.002), and can be interpreted as: when compared to the reference category of ‘no perceived dangers’, an increasing number of perceived dangers is generally reflected in lower odds of having play experiences. For example, for respondents who had perceived one scenario as dangerous, the odds of the children having more play experiences (i.e. five or six experiences versus four or fewer) were 44% lower than if respondents perceived no dangers (odds ratio = 0.56), holding constant all other variables. The odds ratio decreased further to 0.36 and 0.38 for ‘two’ and ‘three or more’ perceived dangers, respectively. From the same model, the odds of children having more play experiences was four times higher for parents (OR = 4.74) compared to practitioners, 4% higher for ever year increase of respondent age, and between 1.8 times to 7.3 times higher as respondent childhood experiences increased (compared to three or less), holding all other variables constant in the model.

In step 2 of the modelling (RQ3), respondents’ ‘perceived number of benefits’ was added to the base model, resulting in perceived dangers no longer demonstrating a significant main effect (Wald test; χ^2^ = 6.04, *p* = 0.11). ‘Perceived benefits’ was a significant main effect (Wald test; χ^2^ = 19.00, *p* < 0.001), and can be interpreted as: when compared to the reference category of ‘four or fewer benefits’, an increasing number of perceived benefits is generally reflected in higher odds of having play experiences. For example, for respondents who had five perceived benefits, the odds of the children having more (i.e. five or six experiences versus four or fewer) play experiences were 1.9 times higher than if respondents perceived four or fewer benefits (odds ratio = 1.91), holding constant all other variables. The odds increased by 3.5 times if respondents recognised benefits in all six play experiences. The other significant main effects from the base model (parent/practitioner, respondent age, and respondent’s childhood experiences) remained stable and significant in this model. The final stage of modelling added whether respondents came from urban or rural locations, and perceived traffic either at home or at the practitioners ELC setting. Neither were significant contributions to the model. All other variables remained unchanged (Table 3).

**Table 3.** Odds ratios from multivariable Ordinal Logistic regression models.

Variable	Step 1 OR (95% CI)	Step 2 OR (95% CI)	Step 3 OR (95% CI)
Perceived danger	ref: None*	–	–
One	0.56 (0.32, 0.97)	0.71 (0.40, 1.27)	0.71 (0.40, 1.26)
Two	0.36 (0.20, 0.66)	0.49 (0.26, 0.90)	0.48 (0.26, 0.90)
Three or more	0.38 (0.20, 0.69)	0.56 (0.29, 1.05)	0.55 (0.29, 1.05)
			
Respondent	ref: Practitioner	–	–
Parent	4.74** (2.77, 8.08)	4.67** (2.74, 7.99)	4.60** (2.68, 7.89)
			
Respondent age	1.04* (1.02, 1.07)	1.05** (1.02, 1.08)	1.05 (1.02, 1.08)
			
Respondent experience	ref: ≤ Three**	**	**
Four	1.76 (0.83, 3.71)	1.78 (0.83, 3.79)	1.79 (0.84, 3.82)
Five	4.54 (2.34, 8.80)	4.08 (2.07, 8.04)	3.92 (1.98, 7.74)
Six	7.26 (3.75, 14.07)	6.51 (3.33, 12.75)	6.65 (3.39, 13.06)
			
Child age group	ref: 2–3 years		
	1.21 (0.76, 1.94)	1.25 (0.78, 2.02)	1.30 (0.80, 2.11)
			
Perceived benefits	–	ref: ≤ Four**	**
Five	–	1.91 (1.06, 3.46)	1.83 (1.00, 3.31)
Six	–	3.48 (1.97, 6.13)	3.44 (1.95, 6.07)
			
Setting	–	–	Ref: Rural
Urban	–	–	1.04 (0.64, 1.61)
			
Perceptions of traffic	–	–	Ref: not dangerous at all
Rather not dangerous	–	–	0.60 (0.28, 1.27)
Dangerous			0.71 (0.33, 1.52)

*P < 0.005, **p < 0.0001

Ref: Reference category

## 5. Discussion

The primary aim of this paper was to explore whether parental/practitioner perceptions of danger and benefits of risky play was associated with their children’s risky play experiences. The headline results support those found in the Belgian study of parents from Jidovtseff et al. (2022): (i) perceived dangers were negatively associated with perceived benefits; (ii) children’s experiences were positively associated with (a) perceived benefits and (b) parent’s own childhood experiences; and (iii) children’s experiences were negatively associated with perceived dangers.

A key analysis in this paper, and indeed contribution to the literature, was the exploration of the potential differential role that being a parent or practitioner may have on perceptions of risky play. However, statistical tests showed that being a parent or practitioner did not moderate the relationship between perceived dangers and children’s outdoor play experiences. Even though the number of play scenarios experienced by their children was higher for parents than practitioners, this was not explained by perceived dangers. Perhaps this reflects parents likely having more time with children to experience a range of risky play scenarios in comparison to practitioners who could be constrained in the number of accessible outdoor play situations, i.e. not all ELC settings may have access to natural elements such as open water, or animals. Additionally, in the context of childcare, professionals sometimes must manage a larger number of children. In such situations, supervision is likely to be more focused on safety, and therefore less permissive when it comes to risky play.

Where practitioner and parental perceptions of danger were significantly associated with children’s risky play experiences these became non-significant when perceived benefits were included in our modelling. So an important finding from this paper is that we must continue to prioritise efforts to raise the awareness of the benefits associated with outdoor play, as these may play an important, and potentially protective, role in determining whether children experience more or less outdoor risky play situations. Where this is combined with actively targeting and reducing the perceived dangers associated with outdoor risky play, this will likely lead to optimal results.

Over the last 15 years we have started to see a more vocal presence of systems thinking in our ‘intervention’ discourse, especially that of Public Health (Carey et al., 2015; Leischow et al., 2008; Peters, 2014) and recently in the optimisation of outdoor nature-based ELC (Zucca et al., 2023) and outdoor play broadly (Cheng et al., 2022; Jerebine et al., 2022b). Ulrich Beck’s foundational text ‘Risk Society: Towards a New Modernity’ (Beck, 1992) frames much of his argument around the societal system of risk created by man; manufactured globally through human activity, and central to government and everyday life. Risk has become a socio-political issue, particularly as to who defines what counts as risk, who is exposed, and who has the power to avoid it. Beck recognised that risk is not just a technical issue, but one deeply entrenched in social phenomenon within modern institutions and our culture. This is as true now as it was in 1992 and poignantly exemplified in our understanding of risky play. As such, the culture and experience of risk in children’s play requires integrated, ecological, approaches involving the child, parent, practitioner and beyond – challenging institutional and societal expectations and norms requires multifaceted approaches at different levels and needs to happen from the bottom up and top down.

Interventions should be informed and underpinned by theory, and researchers should borrow from the abundance of multidisciplinary literature in areas such as ecological/systems theory (Koorts & Rutter, 2021), Parent Development (Mowder, 2005), risk and safety in children’s play (Brussoni et al., 2012) and the fields of Health Psychology and Social Marketing. At an individual level, the Health Psychology literature explores constructs like attitudinal formation. The beliefs, and perceptual positions towards something like risky play, are fundamental to the decision-making processes parents and practitioners make when faced with these situations for the children in their care. Jidovtseff et al. (2022) discussed the value of communication tools and campaigns aimed at parents and the importance of emphasising the benefits whilst contextualising the dangers. Media messaging/campaigns is a powerful tool and has been successfully implemented in Scandinavian countries such as Norway (Sandseter, 2011). Our data and findings support this but should also extend to include practitioners (and other stakeholders) working in early years professions. Considerable innovative work has been conducted over the last five years in the wider physical activity literature (Williamson et al., 2021) with the creation of a theoretically informed process of how to design and develop messaging frameworks targeting social and attitudinal changes, including considerations around the ‘who, when, what, and why’, the ‘message content’, and its ‘format and delivery’. This structured process holds promise for work around risky play and we should borrow much of the messaging template and translate for our parent and practitioner audience.

However, these messages will likely look different depending on context and audience. Natural similarities between a parent and educator exists, and many taking on the role as practitioner or play worker feel like they are operating *in loco parentis* (Connolly & Haughton, 2017; Jerebine et al., 2022b). Our study supports the view that perceptions of danger and benefits in risky outdoor play are not moderated by whether you are a parent or practitioner, however, that does not mean that the conceptualisation of risk, including its value and implications within society, is the same for both. Adapting our approach is imperative. For practitioners, we should delve deeper into the risk literature and engage with the social constructionist perspective to gain greater insight into how structural, societal, and institutional culture of risk aversion, overprotection, and the vulnerability of children’s position in society came to be. We must consider institutional pressures in areas such as the insurance industry that reinforce the risk-aversion approach within school settings. Practitioners involved in a 2023 study exploring the systemic factors involved in optimising outdoor nature-based play and learning frequently raised concerns about litigation, risk assessments, and time-consuming incident report processes for minor issues around outdoor play (Zucca et al., 2023). Regulation and/or litigation, sanctioning, and revocation of licenses to practice have also been raised by educators in Canada (Cheng et al., 2022), and the U.S.A. (LeMasters & Vandermaas-Peeler, 2023) and further supported in a recent systematic review reflecting similar concerns more widely (Jerebine et al., 2022b). This can lead to restrictive policies being implemented by ELC settings. Connolly and Haughton (2017) demonstrated this in Welsh educators: a minority of participants within their study considered tree climbing to be too risky and banned it completely, citing institutional policy. These are echoed in other countries such as the United States where examples of ‘red tape’ or lack of supportive school policy prevents outdoor play (e.g. Ren & Langhout, 2010). In Scotland, we are fortunate to see significant changes in the ELC policy environment and regulatory risk framework with standards of care bodies such as the Care Inspectorate – who inspect and register our ELC practices in Scotland – launching a position statement in 2016 supporting a *positive approach to risk to achieve the best outcomes for children. This means moving away from a traditional deficit model that takes a risk-averse approach …  … .to a more holistic risk-benefit model* (Care Inspectorate, 2016). With the recent introduction of the new International Standard (ISO 4980; International Organization for Standardization, 2023) ‘Benefit-risk assessment for sports and recreational facilities, activities and equipment’ that echoes this position, the culture of risk management, including (concerns over) litigation may change. Against a backdrop of the United Nations Convention on the Rights of the Child (UNCRC; United Nations, 1989), including its Article 31, colloquially termed the ‘Right to Play’, hopefully being incorporated into Scots law, Scotland is taking up the challenge of implementing systems change (Scottish Government, 2023).

Practitioner perceptions of risk are clearly impacted, and shaped, by the wider professional setting in which they work. That, however, does not necessarily reflect how they may parent, nor how they perceive the dangers or benefits associated with risky play thinking as a parent. Five out of six scenarios presented to parents (all excluding that involving rain) in our study had over 10% reporting as dangerous with no benefit (compared to two out of five for practitioners), with concerns over ‘safety’ a well cited issue underpinning those perceptions of dangers. In each of the six scenarios the common thread was a concern about personal physical injury (slipping, falling, hurting themselves), echoing international evidence from a 2022 systematic review (Jerebine et al., 2022b). However, these issues may be culturally specific; Sandseter and colleagues, for instance, demonstrated that fear of injury was not raised as a concern by any parents in Estonia and Norway, supporting the need to tailor our intervention efforts to the specific type of play but also the country/culture of the setting (Sandseter et al., 2020). In addition to more contextually focused messaging of the known benefits of risky play, further intervention development work should be conducted with parents to explore how to prioritise our actions in the short, medium and long term.

With our findings demonstrating that parental childhood experiences are significantly associated with perceptions of dangers and childhood experiences, there is a strong chance that parents themselves do not feel confident in either supporting outdoor play or risk. Jidovtseff et al. (2022) spoke of the potential intergenerational consequences and the vicious cycle that may ensue from parents-to-be having less experiences of risky play, subsequent elevated levels of perceived dangers, and fewer experiences for their own children. Simple practices where ELC settings actively intervene to improve outdoor play opportunities for their children, whilst simultaneously supporting parents with exposure and positive messaging, may provide small steps to challenging and changing the negative cultural norms around risky play.

## 6. Conclusions

### 6.1. Future directions

Context is important – the same situations are perceived to be more dangerous in some places than others. For instance, 93% of respondents in the current analysis said that there was both benefit and no danger associated with playing outdoors in the rain. Not considered risky under the original classification by Sandseter (2010), it was added to the survey to reflect the often-cited barrier that is poor weather. In Scotland this might be rain, whereas in other parts of the world this barrier may be reflected in intolerable heat, sun damage, and/or freezing temperatures. A continuation of culturally specific evidence will be valuable for researchers exploring intervention opportunities. Most cited benefits are also culturally and context specific, with implications for how interventions are developed. For example, Vandermaas-Peeler et al. (2019) compared US and Danish parents’ perceptions of the benefits of children’s play and nature experiences and found that US parents focused on the benefits associated with child development, whereas Danish parents also recognised the societal and environmental benefits of children being connected with play in nature. Parents in the present study cited benefits ranging from improvement in motor skills, social relationships, autonomy, utilitarian value, and nature connection. With internationally varied evidence in this area improving, it is becoming clearer that tailored approaches (Sandseter et al., 2020), that reflect the expectations and values of the context and culture, are required when targeting constructs such as benefits of outdoor/nature play.

### 6.2. Strengths and limitations

The design of this study closely followed that of previously published work in a Belgian cohort (Jidovtseff et al., 2022). With methodological transparency from the original authors, we were able to replicate the approach in a Scottish context with comparable findings. We add to this literature base by including the perceptions of practitioners and formally testing the moderating role being a parent or practitioner may have had. However, with the design of this study being cross-sectional and observational, we are unable to evaluate the bi-directional nature of the relationships. For example, the results from this paper support the position that perceived dangers have a negative association with the risky play experiences of children, whereas perceived benefits support children’s experiences of risky play. The alternative could be equally true: the greater number of risky play situations that children experience, the lower number of parent/practitioner perceived dangers, and the higher number of perceived benefits. It could be argued that children’s initial experiences of risky play are the result of exposure that is gatekept by adults. With positive experiences these then introduce or reinforce the benefits and reduce the perceived dangers.

Some critique can be aimed more generally at image-based survey methodology, and the inherent issue of image choice as a strong determinant of respondent perception (Couper et al., 2004). This was minimised by integrating a preparatory stage in the research design whereby an independent group of parents assisted with image choice and accompanying text. Issues were clarified and changed where required and images ultimately chosen to best reflect the cultural context of Scotland. The accompanying text alongside the image is an addition that reduces bias in the interpretation as it provides context and framing for how the image should be interpreted. For our analyses we were unable to separate by parent/practitioner gender due to majority female respondents and we did not stratify our analyses by child gender. The former is particularly important as male caregivers have been reported to have higher propensity for risk in themselves and for their children (Duflos et al., 2023). Both could be explored by researchers in future work. Finally, because it was important to compare images between parents and practitioners that were conceptually similar, we were only able to combine six of the outdoor scenarios of the original ten so our analyses have removed some of the, arguably, higher danger situations such as using a tools (e.g. a saw). The inclusion of these types of scenarios could, conceivably, have resulted in differences between respondents, but further work would need to be conducted to test this.

### 6.3. Final thoughts

Perceptions of danger associated with outdoor (risky) play scenarios were relatively low in this sample of Scottish parents and ELC practitioners. Perceptions of the benefits associated with these same scenarios were encouragingly high and this was reflected in the average number of play scenarios children experienced within the sample. In support of the wider literature, perceived dangers were negatively associated – and perceived benefits positively associated – with children’s play experiences. Our regression modelling demonstrated that perceived benefits may nullify the effects of perceived dangers, with no moderation by whether respondents were parents or practitioners. We contribute to the evidence base by positioning this work within a Scottish geographical and cultural context, and by including both parents and practitioners within our sample.

If we aspire to a lifecourse prevention model of public health, whereby early intervention can support and maintain positive health and wellbeing in later life, we must strengthen our understanding of the determinants that may contribute to the impact and, ultimately, success of any intervention. To optimise our endeavours, we must employ systems thinking that recognises the complexity of cultural change, and the interdependencies between and within the multiple spheres of influence that impact children’s experiences of risky play – the home and ELC settings being vitally important behavioural contexts. If we are to change the minds of every Henry Brown (featured in Paddington) parent that prevents their child from jumping up and off a train station bench, then promoting the benefits of risky play is a good place to start. Positioning this within a wider approach of influencing and lobbying institutional change, including national level policy and guidance on managing risk; local level action such as ELC leadership on the integration of a risk-benefit model; and 100% support to practitioners and parents to implement opportunities to engage with risk, then we will, with time, hopefully see a gradual shift in the way children experience and exercise their right to play.

## Data Availability

Anonymised data will be made available to share via the University of Glasgow Enlighten repository: Parents - 10.5525/gla.researchdata.1245, Practitioners - 10.5525/gla.researchdata.1246. Please contact the corresponding author for further details.
